# Unraveling malignant phenotype of peritumoral tissue: transcriptomic insights into early-stage breast cancer

**DOI:** 10.1186/s13058-024-01837-2

**Published:** 2024-06-03

**Authors:** Pere Miquel Morla-Barcelo, David Laguna-Macarrilla, Octavi Cordoba, Gabriel Matheu, Jordi Oliver, Pilar Roca, Mercedes Nadal-Serrano, Jorge Sastre-Serra

**Affiliations:** 1 Grupo Multidisciplinar de Oncología Traslacional, Institut Universitari d’Investigació en Ciéncies de la Salut (IUNICS), Universitat de les Illes Balears, Palma, Illes Balears, Spain; 2 Instituto de Investigación Sanitaria de las Islas Baleares (IdISBa), Hospital Universitario Son Espases, Edificio S, Palma, Illes Balears, Spain; 3 Departamento de Patología, Hospital Universitari Son Espases, Palma, Illes Balears, Spain; 4 Servicio de Obstetricia y Ginecología, Hospital Universitari de Son Espases, Palma, Illes Balears, Spain; 5 Facultat de Medicina, Universitat de les Illes Balears, Palma, Illes Balears, Spain; 6 CIBER Fisiopatología Obesidad y Nutrición, Instituto Salud Carlos III, Madrid, Spain

**Keywords:** Breast cancer, Early-stage, Peritumoral tissue, Relapse, Transcriptomics, Biomarkers

## Abstract

**Background:**

Early-stage invasive ductal carcinoma displays high survival rates due to early detection and treatments. However, there is still a chance of relapse of 3–15% after treatment. The aim of this study was to uncover the distinctive transcriptomic characteristics and monitoring prognosis potential of peritumoral tissue in early-stage cases.

**Methods:**

RNA was isolated from tumoral, peritumoral, and non-tumoral breast tissue from surgical resection of 10 luminal early-stage invasive ductal carcinoma patients. Transcriptome expression profiling for differentially expressed genes (DEGs) identification was carried out through microarray analysis. Gene Ontology and KEGG pathways enrichment analysis were explored for functional characterization of identified DEGs. Protein-Protein Interactions (PPI) networks analysis was performed to identify hub nodes of peritumoral tissue alterations and correlated with Overall Survival and Relapse Free Survival.

**Results:**

DEGs closely related with cell migration, extracellular matrix organization, and cell cycle were upregulated in peritumoral tissue compared to non-tumoral. Analyzing PPI networks, we observed that the proximity to tumor leads to the alteration of gene modules involved in cell proliferation and differentiation signaling pathways. In fact, in the peritumoral area were identified the top ten upregulated hub nodes including CDK1, ESR1, NOP58, PCNA, EZH2, PPP1CA, BUB1, TGFBR1, CXCR4, and CCND1. A signature performed by four of these hub nodes (CDK1, PCNA, EZH2, and BUB1) was associated with relapse events in untreated luminal breast cancer patients.

**Conclusions:**

In conclusion, our study characterizes in depth breast peritumoral tissue providing clues on the changes that tumor signaling could cause in patients with early-stage breast cancer. We propose that the use of a four gene signature could help to predict local relapse. Overall, our results highlight the value of peritumoral tissue as a potential source of new biomarkers for early detection of relapse and improvement in invasive ductal carcinoma patient’s prognosis.

**Supplementary Information:**

The online version contains supplementary material available at 10.1186/s13058-024-01837-2.

## Background

Invasive ductal carcinoma (IDC) is the most common type of breast cancer and improvements in early detection and treatment have resulted in high survival rates [1, 2]. However, a fraction of patients suffers a relapse within five years, highlighting the ongoing challenge of managing breast cancer recurrence [3]. To address this issue, the efforts are focused on identifying targets that can detect and delay relapse, improving prognosis [4, 5]. As is well known, the standard of care for patients with early-stage breast cancer is lumpectomy or mastectomy alone or combined with radiotherapy [6]. However, although patients with advanced-stage breast cancer are more likely to develop a relapse than patients with early-stage breast cancer, it has been observed that after surgical and radiotherapy treatment, approximately 3–15% of woman with early-stage breast cancer experience a local recurrence within 10 years after treatment [3].

Historically, it was thought that the main cause of local recurrence after these treatments is primarily due to residual infiltration of tumor cells in normal tissues beyond the assessed surgical margin [7]. However, new findings and further characterization of non-tumoral tissue close to the tumor, known as peritumoral tissue, have revealed significant alterations that suggest the peritumoral tissue as a distinct entity that may have important implications for cancer screening and prevention approaches [8, 9]. These findings challenge the conventional view and suggest that peritumoral tissues may provide crucial information that goes beyond that obtained from the tumor itself. In this context, the study of the tumor microenvironment theory and the field cancerization theory proposed by Slaughter et al. has acquired an impulse to understand the recurrent tumors and pre-malignant changes [10–12].

In breast cancer, the field cancerization theory refers to the spread of genetic and epigenetic changes in normal breast tissue surrounding tumor that may contribute to breast cancer relapse [11]. In this scenario, most research is focused on genetic mutations and molecular changes in peritumoral tissue to develop novel strategies to eliminate visible tumors and prevent the development of secondary cancers [13–16]. Therefore, recent investigations, focused on understanding this process, are revealing the essential role of pre-malignant tissue study in improving early detection and targeted therapies [5, 11].

Taking this evidence together, this study was designed to understand the distinctive characteristics of peritumoral tissue in invasive ductal carcinoma, specifically in a luminal molecular subtype early-stage cohort, and to examine its potential as a valuable target for monitoring recurrence and improving prognosis. The study of patients with early-stage breast cancer would also provide insights into the first changes that occur in the progression of the tumor to advanced stages. Therefore, monitoring the observed changes in peritumoral tissue in the remaining breast gland non-tumoral tissue after surgical resection could contribute to the early detection of relapse and improve the prognosis and management of patients.

## Methods

### Patients and tissue sample

This study was performed in a cohort of 10 patients who were surgically treated for early-stage invasive ductal carcinoma (IDC) during 2020–2022 at the Hospital Universitari de Son Espases (HUSE), Balearic Islands, Spain. All primary tumors present a luminal molecular subtype distributed in early-stages I (*n* = 7) and II (*n* = 3). All patients signed the informed consent according to the World Medical Association Declaration of Helsinki that medical research involves human subjects and were informed of the study project, approved by the Balearic Islands Bioethics Committee (IB4558-21PI). Tissue samples included in the study were histologically classified by a pathologist and stored in RNAlater® (AM7021, Fisher Scientific, Madrid, Spain) at -80 °C immediately. Tumoral (TT), Peritumoral (PT), and Non-tumoral (NT) breast tissue samples from each patient were used for gene expression profiling. PT samples were identified as non-neoplastic tissue located approximately 1.5 cm away from the tumor lesion. To confirm the histological findings and validate the identification of PT, paraffin sections were prepared and examined by a pathologist. Specifically, the presence of ductal carcinoma in situ (DCIS) or epithelial atypia at the periphery of the tumor was evaluated. Patients had a mean age of 60.1 years (SD: 13.8 and range: 28–76). The clinicopathological characteristics of the patients enrolled in the study are shown in Table S1.

### Tissue Homogenization and RNA extraction

Total RNA was isolated from tissue samples (40–100 mg) by TriReagent® extraction (T9424, Sigma-Aldrich, St. Louis, MO, USA) according to the manufacturer’s instructions. Tissue samples were homogenized using a polytron homogenizer (T10 basic, IKA -Werke 6 mbH, Staufen, Germany) in TriReagent®. RNA isolated was resuspended in RNAase-free water to be further used for microarray analysis. RNA quality and concentration were determined using the BioSpecnano spectrophotometer (Shimadzu Biotech, Kyoto, Japan). Additionally, RNA integrity was also assessed with the Agilent 2100 Bioanalyzer (Agilent Technologies, USA).

### Microarray-based transcriptome profiling

Fifty ng total RNA was reverse transcribed following the instructions of the GeneChip 3’IVT Pico Kit (#902,789, Thermo Fisher Scientific, Madrid, Spain). Briefly, complementary RNA (cRNA) was generated by using low-cycle PCR amplification and subsequent T7 in vitro transcription. After purification, double-stranded cDNA was synthesized by a combination of reverse transcription of cRNA and subsequent DNA polymerization of the sense-strand cDNA. The cRNA template was then hydrolyzed, and 5.5 µg of purified ds-cDNA was used for fragmentation, biotin-labeling, and hybridization to Clariom S human transcriptome arrays (#902,926, Thermo Fisher Scientific, Madrid, Spain) at 45 °C for 17 h using the GeneChip 645 hybridization oven. The arrays were washed, and stained on the FS450 Fluidics Station, and scanned using the GeneChip Scanner 3000 7G (Thermo Fisher Scientific, Madrid, Spain) according to the manufacturer’s protocol. Several quality controls were introduced into the experimental workflow according to the manufacturer’s protocol to check RNA quality, probe synthesis, and hybridization performance. Therefore, from all samples, raw CEL files of 9 TT, 7 PT, and 8 NT samples were extracted and analyzed.

### Differential expression profiling of the transcriptome

Differentially expressed genes (DEGs) were identified by comparison of TT, PT, and NT by using ThermoFisher Transcriptome Analysis Console (TAC) using SST-RMA normalization and summarization methods. The criteria for selecting DEGs as significantly differentially expressed genes were a |Fold Change (FC)|≥2 and a p-value ≤ 0.05. From all tissue comparisons, DEGs were then divided into those that were upregulated (FC ≥ 2) and downregulated (FC≤-2). Additionally, the volcano plots of each comparison and the hierarchical clustering of the samples were obtained from the TAC software.

Expression of the most relevant hub nodes was validated using GSE72644 database. Information on the transcriptomic alterations of tumoral tissue and the duct leading to the tumor (proximal and distal) from luminal breast cancer patients is available in this database. Microarray expression intensities plots were analyzed using Student’s t-test.

### Gene set enrichment analysis

DEGs with |Fold Change (FC)|≥1 and a p-value ≤ 0.05 were used to acquire Normalized Enrichment Scores (NES) of Gene Ontology (GO) and Kyoto Encyclopedia of Genes and Genomes (KEGG) pathways using Gene Set Enrichment Analysis (GSEA) method of WEB-based GEne SeT AnaLysis Toolkit (WebGestalt). The enrichment analysis of cancer-specific Hallmark Gene Sets was developed using GSEA software [17, 18]. Only gene sets with a p-value ≤ 0.05 and an FDR value ≤ 0.05 were considered significantly enriched.

### Protein-protein interaction network analysis

Network analysis using the online tool ‘NetworkAnalyst’23 based in the Search Tool for the Retrieval Interacting Genes (STRING) interactome as a database was performed [19]. The confidence cutoff score was set to 900. Protein-Protein Interaction (PPI) networks were constructed with first-order interaction network analysis, considering direct and indirect interactions between DEGs with a |Fold Change (FC)|≥2 and a p-value ≤ 0.05 (presented as nodes). Node size is positively associated with degree, and the thickness of the edge is correlated with the connections among proteins. The main functional modules of the networks were selected using the WalkTrap algorithm and functionally characterized by KEGG pathways enrichment analysis using ‘NetworkAnalyst’23 tool. P-value ≤ 0.05 was considered significantly enriched.

### Overall survival and relapse-free analysis

Overall Survival (OS) and Relapse-Free Survival (RFS) analysis of breast cancer patients were determined using cBioPortal software [20–22]. From all breast cancer microarray mRNA expression of METABRIC database, estrogen and progesterone receptor positive patients without endocrine and chemotherapy treatment were taken. Prognosis was evaluated comparing the patients with high and low individual expression of the most relevant PPI network hub nodes (patients divided by median). OS and RFS analysis of the gene signature were assessed and high/low expression groups were defined based on the median expression values (genes equally weighted). Particularly, high and low expression groups were defined as samples with values above and below, respectively, the median expression value for all four genes. Hazard ratios with 95% confidence intervals were noted and a LogRank p-value ≤ 0.05 was considered statistically significant.

### Real-time qPCR

RT-qPCR was used to confirm gene expression levels of the four-gene signature. For each RT-qPCR validation, cDNA was obtained by retrotranscription, and PCR reactions were carried out as previously reported [23]. The relative quantity of each gene was determined for each sample, and the relative quantity of each test gene was calculated after normalization with 18 S. The cycle threshold (Ct) values obtained from real-time PCR were analyzed, considering the reaction efficiency and normalizing these results to GAPDH, using the GenEx Standard Software (MultiDAnalises, Sweden). Genes, their corresponding primers, and annealing temperatures are shown in Supplementary Table S2.

### Immunohistochemistry analysis

For immunohistochemical (IHC) corroboration of the microarray data, 5-µm serial sections were cut from formalin-fixed paraffin-embedded (FFPE) samples, deparaffinized in CLEAR Histo 775 (AF-21His775, CasaÁlvarez, Madrid, Spain) and rehydrated in graded alcohol to water. The slides were steamed in 10 mM Citrate Buffer (NB-23-00174, NeoBiotech, Nanterre, France) for 20 min and endogenous peroxidase was blocked for 15 min with 3% H_2_O_2_ solution. Slides were blocked with 5% Bovine Serum Albumine (Sigma-Aldrich, St. Louis, MO, USA) 0.1% Triton X100 (T8787, Sigma-Aldrich, St. Louis, MO, USA) in 1X PBS for 60 min at room temperature. The primary antibody of CDK1 (1:200, 19532-1-AP, Proteintech®), PCNA (1:200, 24036-1-AP, Proteintech®), EZH2 (1:400, 66476-1-Ig, Proteintech®), and BUB1 (1:400, 13330-1-AP, Proteintech®) were incubated overnight at 4 °C. Slides were then reacted with goat anti-rabbit and goat anti-mouse biotinylated secondary antibody (BA-1000 and BA-9200, Vector Laboratories, Newark, CA, USA) for 60 min and incubated with HRP-Streptadivine solution (NB-23-00001-4, NeoBiotech, Nanterre, France). Diaminobenzidine (ACB030, ScyTek Laboratories, Logan, UT, USA) was used as a substrate. Sections were counterstained with hematoxylin (GHS116, Sigma-Aldrich, St. Louis, MO, USA), dehydrated, and mounted. Images were acquired and analyzed using a Nikon Eclipse 50i microscope.

## Results

### Identification of DEGs in early-stage invasive ductal carcinoma tissue samples

**Fig. 1:**
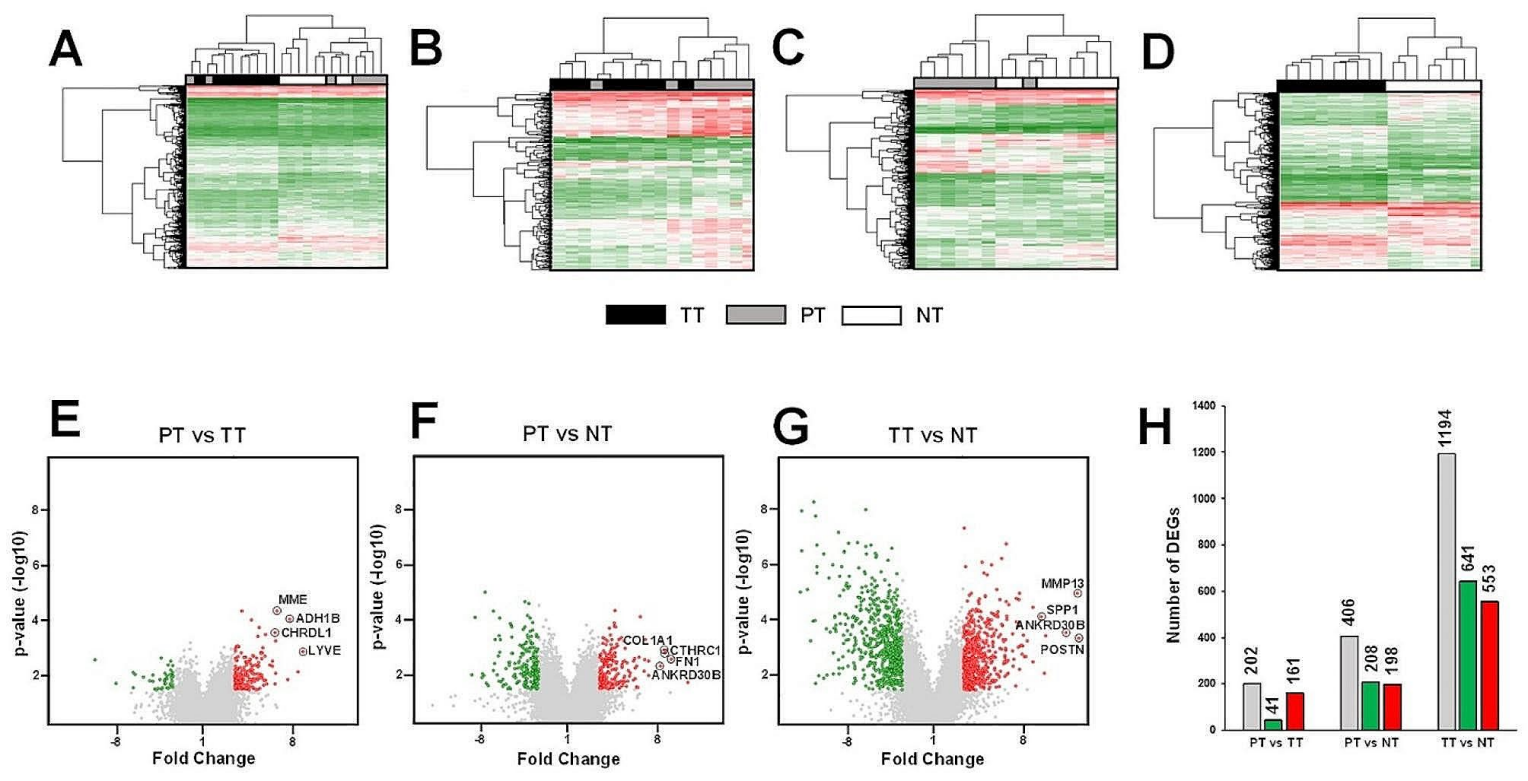
Hierarchical clustering and DEGs identification in early-stage IDC samples. Heat map showing the differential gene expression profiles between TT, PT and NT samples (**A**) as well as the differences in gene expression in PT compared TT (**B**), PT samples compared to NT (**C**) and TT samples compared to NT (**D**). Each column represents one sample. Each row represents a single gene: green denotes a low relative expression while red denotes a high relative expression; Volcano plot displaying DEGs of PT samples compared to TT (**E**) or NT (**F**) samples as well as the differences in gene expression between TT and NT (**G**). Green dots and red dots represent downregulated and upregulated DEGs, respectively; (**H**) Bar diagram showing the number of identified downregulated and upregulated DEGs: gray bars are used to represent all DEGs while green and red are used to represent downregulated and upregulated DEGs, respectively

Transcriptome expression profiling was performed by using microarray to find out the molecular changes in Tumoral (TT), Peritumoral (PT) and Non-tumoral (NT) tissues of early-stage invasive ductal carcinoma (IDC). The hierarchical clustering analysis indicated that TT, PT, and NT samples had a clear aggrupation when the three tissues were compared (Fig. 1A). The heat map of PT compared to TT (Fig. 1B) or NT samples (Fig. 1C), as well as the comparison between TT and NT (Fig. 1D), evidenced the distinctive profile of both PT vs. TT and PT vs. NT.

Differentially Expressed Genes (DEGs) were identified with a cutoff of |FC|≥2 and p-value ≤ 0.05. Thus, the integral variation of gene expression in the three comparisons were represented by volcano plots (Fig. 1E-G). As shown in Fig. 1H, a total of 202 DEGs (41 downregulated and 161 upregulated) and 406 DEGs (208 downregulated and 198 upregulated) were identified in PT compared to TT and NT, respectively. Whereas 1194 DEGs (641 downregulated and 553 upregulated) were found in TT compared to NT.

As shown in Table 1, the ten most downregulated DEGs in PT versus TT comparison were CST1, CST4, PGR, SPP1, MMP13, GJB2, COL11A1, CLDN7, HIST1H2BD and S100A14, and the ten most upregulated DEGs were LYVE, FOSB, ADH1B, ABCB5, MME, CHRDL1, FHL5, SRPX, ACKR1, and ANK2. On the other hand, in PT versus NT comparison, the most significantly downregulated DEGS were CNN1, VIT, LEP, NTRK2, PTN, FXYD1, SAA1, SAA2, FIGF and KLK5, and the most upregulated DEGs were POSTN, FN1, CTHRC1, COL1A1, ANKRD30B, PRLR, COL5A1, TOP2A, SERPINE1, and MMP13. Finally, KRT14, LYVE1, VIT, CRYAB, SFRP1, NTRK2, LEP, MME, RBP4, and PIK3C2G were the top ten downregulated DEGs, and POSTN, MMP13, ANKRD30B, SPP1, HIST1H2BM, GJB2, FN1, TSPAN1, TRPS1, and CST1 were the top ten upregulated in TT samples compared to NT.

**Table 1:** Top ten downregulated and upregulated DEGs of tissue comparisons

PT vs. TT			PT vs. NT			TT vs. NT		
Gene	FC	*p*-value	Gene	FC	*p*-value	Gene	FC	*p*-value
**Downregulated genes**								
CST1	-11.64	0.0037	CNN1	-8.99	0.015	KRT14	-32.4	0.016
CST4	-7.23	0.028	VIT	-8.38	9.87E-05	LYVE1	-19.61	1.03E-05
PGR	-4.95	0.042	LEP	-7.18	0.0184	VIT	-19.19	3.00E-07
SPP1	-4.94	0.0112	NTRK2	-7.04	0.013	CRYAB	-19.07	9.56E-09
MMP13	-4.73	0.013	PTN	-6.83	0.0156	SFRP1	-16.84	8.65E-06
GJB2	-3.93	0.016	FXYD1	-6.64	1.13E-05	NTRK2	-15.68	0.0007
COL11A1	-3.39	0.0223	SAA1	-6.42	0.0304	LEP	-14.69	0.0001
CLDN7	-3.17	0.0238	SAA2	-6.21	0.0376	MME	-14.54	4.34E-09
HIST1H2BD	-3.16	0.0137	FIGF	-5.51	5.89E-05	RBP4	-14.11	0.0001
S100A14	-3.03	0.0464	KLK5	-5.48	0.0097	PIK3C2G	-13.98	0.0283
**Upregulated genes**								
LYVE	9.22	0.0018	POSTN	14.62	0.0287	POSTN	26.53	0.0006
FOSB	8.24	0.0107	FN1	10.11	0.0038	MMP13	25.77	1.15E-05
ADH1B	6.82	0.0001	CTHRC1	8.74	0.0021	ANKRD30B	20.04	0.0003
ABCB5	6.53	0.0199	COL1A1	8.69	0.0017	SPP1	12.72	0.0005
MME	5.16	5.58E-05	ANKRD30B	7.89	0.0067	HIST1H2BM	12.21	0.0119
CHRDL1	5.01	0.0007	PRLR	6.04	0.0045	GJB2	11.42	9.18E-05
FHL5	4.97	0.0119	COL5A1	6.02	0.0154	FN1	9.48	3.84E-05
SRPX	4.94	0.0004	TOP2A	5.64	0.0007	TSPAN1	9.44	0.0044
ACKR1	4.9	0.0292	SERPINE1	5.55	0.0039	TRPS1	8.37	0.0007
ANK2	4.64	0.0063	MMP13	5.45	0.0106	CST1	8.1	0.0028

TT: Tumoral Tissue, PT: Peritumoral Tissue, NT: Non-tumoral Tissue, and FC: Fold change

### Functional characterization of differential expressed genes

Gene Set Enrichment Analysis (GSEA) software was used to analyze Kyoto Encyclopedia of Genes and Genomes (KEGG) pathway enrichment and the Gene Ontology (GO) of the all DEGs with a |Fold Change (FC)|≥1 and a p-value ≤ 0.05.

**Fig. 2:**
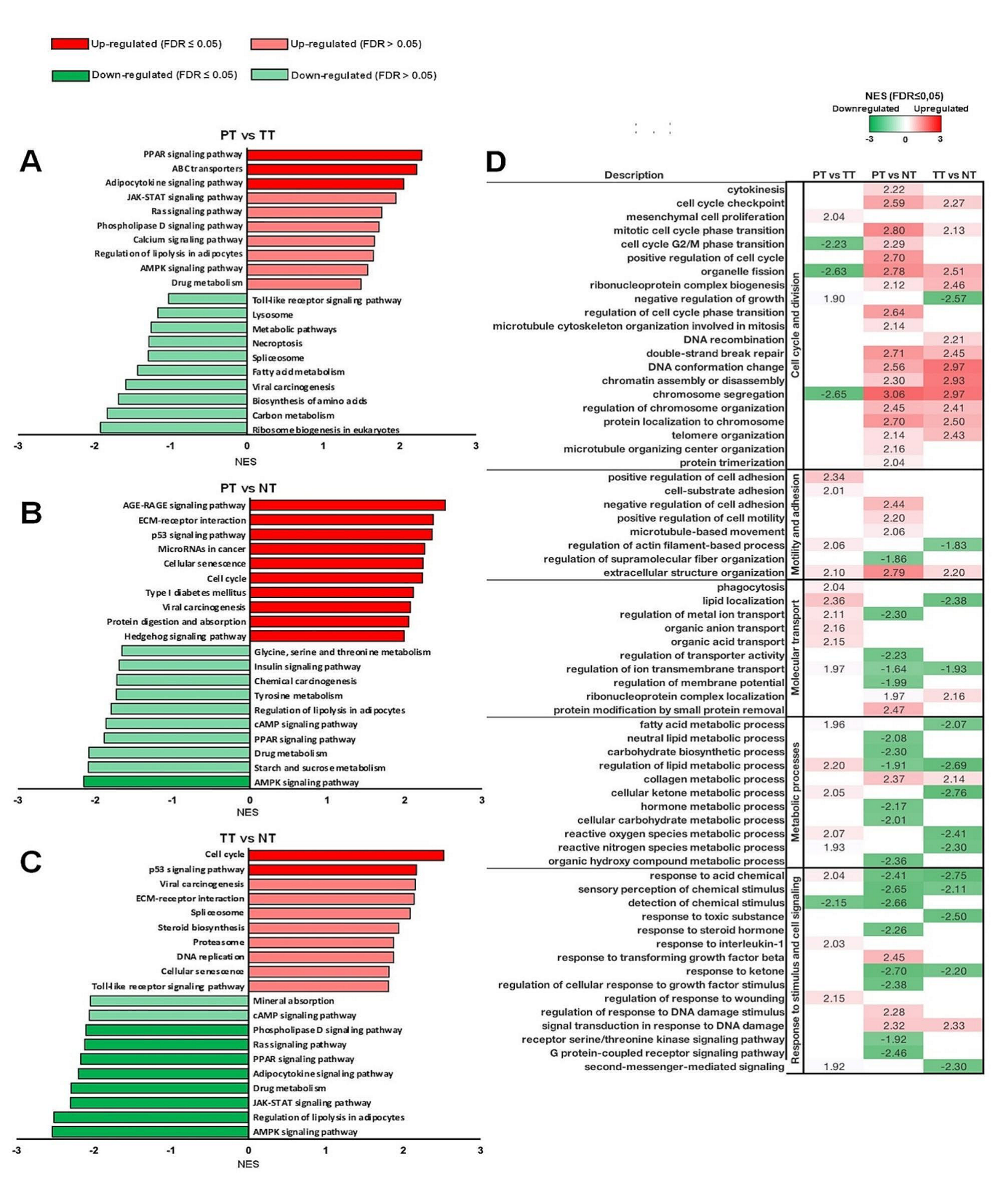
Identification of signaling pathways alterations in early-stage IDC samples. KEGG functional enrichment was performed using GSEA with identified DEGs in PT compared to TT (**A**) or NT (**B**), and TT compared to NT (**C**). Green and red bars show the negative and positive NES values of each KEGG pathway, respectively. Pathways with FDR value ≤ 0.05 are represented by dark bars. Functional enrichment analysis with clustered Biological Processes GO terms were performed using GSEA with DEGs identified in PT compared to TT or NT, and TT compared to NT **(D).** Green cells and red cells show the negative and positive NES values of each GO term, respectively. Representative GO terms are shown (FDR value ≤ 0.05)

On the one hand, the analysis comparing PT versus TT (Fig. 2A) revealed significant upregulation in pathways related to metabolism (PPAR signaling, ABC transporters, Adipocytokine signaling, AMPK signaling), signaling cascades (JAK-STAT, Ras), and cellular responses (Calcium signaling, Regulation of lipolysis). Conversely, PT exhibited downregulation in pathways associated with biosynthesis (Ribosome biogenesis, Amino acid biosynthesis), and metabolism (Fatty acids, Spliceosome). In contrast, the comparison between PT and NT (Fig. 2B) exhibited enrichment in pathways related to AGE-RAGE signaling, ECM-receptor interaction, and cellular responses (p53 signaling, Cellular senescence). Conversely, PT showed decreased activity in pathways associated with basic cellular processes (Ribosome biogenesis, Amino acid biosynthesis) and metabolic functions (Fatty acid metabolism, Metabolic pathways).

On the other hand, the comparison between TT and NT (Fig. 2C) demonstrated activation of pathways linked to cell growth and DNA integrity (Cell cycle, p53 signaling, DNA replication) and extracellular interactions (ECM-receptor, Viral carcinogenesis). Notably, TT displayed reduced activity in metabolic and adipocyte-related pathways (AMPK, PPAR signaling, Regulation of lipolysis) compared to NT.

Biological processes (Fig. 2D) carried out by DEGs were analyzed to further explore the enriched pathways shown previously. Significant DEGs of PT and TT compared to NT exhibited a positive NES value for most GO terms clustered in Cell cycle and cell division processes. Nevertheless, when comparing PT to TT, *mesenchymal cell proliferation* and *negative regulation of growth* GO terms exhibited a positive NES, while *cell cycle G2/M phase transition*, *organelle fission*, and *chromosome segregation* GO terms showed a negative NES.

Furthermore, while PT showed positive NES values in GO terms *positive regulation of cell adhesion, cell-substrate adhesion*, and *regulation of actin filament-based process* compared to TT, displayed a positive NES in *negative regulation of cell adhesion*, *positive regulation of cell motility*, and *microtubule-based movement* GO terms compared to NT. *Extracellular structure organization* GO term displayed a unique pattern in this cluster with a positive NES in PT compared to both TT and NT.

Most GO terms clustered in Molecular transport, Metabolic processes, and Response to stimuli and cellular signaling showed a positive NES in PT compared to TT. In these biological processes, although PT exhibited a negative NES value in most GO terms when compared to NT, GO terms of Response to stimulus and cell signaling cluster *response to transforming growth factor beta*, *regulation of response to DNA damage stimulus*, and *signal transduction in response to DNA damage* showed a positive NES value.

Additionally, in the enrichment analysis of cancer-specific Hallmark Gene Sets (Table 2), PT exhibited a statistically significant negative NES for the *G2M_CHECKPOINT* Hallmark gene get compared to TT, while showing statistically significant positive NES values for hallmark gene sets associated with inflammation and hormonal response. Conversely, similar to TT, PT compared to NT showed statistically significant positive NES values for hallmark gene sets related to epithelial-mesenchymal transition, inflammation, and cell cycle.

**Table 2:** Enrichment analysis of Hallmark gene sets in tissue comparisons

	PT vs. TT		PT vs. NT		TT vs. NT	
Gene Set	NES	*p*-value	NES	*p*-value	NES	*p*-value
*HALLMARK_ADIPOGENESIS*	**2.09**	**0.00**	**-2.67**	**0.00**	**-2.85**	**0.00**
*HALLMARK_ALLOGRAFT_REJECTION*	0.74	0.79	**2.35**	**0.00**	**1.68**	**0.03**
*HALLMARK_ANDROGEN_RESPONSE*	**1.71**	**0.02**	1.30	0.19	1.45	0.10
*HALLMARK_ANGIOGENESIS*	-	-	**2.23**	**0.00**	**2.60**	**0.00**
*HALLMARK_APOPTOSIS*	**1.70**	**0.02**	**1.75**	**0.04**	-1.24	0.22
*HALLMARK_COAGULATION*	**1.74**	**0.02**	**1.81**	**0.02**	-1.26	0.20
*HALLMARK_COMPLEMENT*	0.79	0.69	**2.02**	**0.00**	**2.09**	**0.00**
*HALLMARK_DNA_REPAIR*	**-2.01**	**0.01**	1.58	0.06	**1.96**	**0.01**
*HALLMARK_E2F_TARGETS*	**-2.68**	**0.00**	**3.08**	**0.00**	**3.33**	**0.00**
*HALLMARK_EPITHELIAL_MESENCHYMAL_TRANSITION*	-1.37	0.13	**3.48**	**0.00**	**2.78**	**0.00**
*HALLMARK_ESTROGEN_RESPONSE_EARLY*	**-1.86**	**0.01**	**1.87**	**0.02**	**3.04**	**0.00**
*HALLMARK_ESTROGEN_RESPONSE_LATE*	-1.46	0.09	**2.34**	**0.00**	**2.61**	**0.00**
*HALLMARK_G2M_CHECKPOINT*	**-2.68**	**0.00**	**3.08**	**0.00**	**3.14**	**0.00**
*HALLMARK_HEDGEHOG_SIGNALING*	-	-	**-1.64**	**0.04**	-1.39	0.14
*HALLMARK_HYPOXIA*	**1.78**	**0.01**	1.42	0.10	**-1.84**	**0.00**
*HALLMARK_INFLAMMATORY_RESPONSE*	1.24	0.19	**1.97**	**0.01**	1.40	0.11
*HALLMARK_INTERFERON_ALPHA_RESPONSE*	0.72	0.79	**2.31**	**0.00**	**2.10**	**0.00**
*HALLMARK_INTERFERON_GAMMA_RESPONSE*	1.02	0.43	**2.34**	**0.00**	1.21	0.25
*HALLMARK_MITOTIC_SPINDLE*	-1.21	0.22	**2.86**	**0.00**	**2.25**	**0.00**
*HALLMARK_MTORC1_SIGNALING*	-1.21	0.22	**1.99**	**0.01**	**2.35**	**0.00**
*HALLMARK_MYC_TARGETS_V1*	**-1.72**	**0.03**	**2.35**	**0.00**	**2.81**	**0.00**
*HALLMARK_MYC_TARGETS_V2*	**-1.76**	**0.02**	-	-	**2.38**	**0.00**
*HALLMARK_MYOGENESIS*	**2.19**	**0.00**	**-2.71**	**0.00**	**-2.74**	**0.00**
*HALLMARK_PI3K_AKT_MTOR_SIGNALING*	-	-	**1.64**	**0.05**	1.34	0.16
*HALLMARK_REACTIVE_OXYGEN_SPECIES_PATHWAY*	-	-	**-1.53**	**0.05**	-1.29	0.22
*HALLMARK_TGF_BETA_SIGNALING*	-	-	**2.30**	**0.00**	**1.90**	**0.02**
*HALLMARK_TNFA_SIGNALING_VIA_NFKB*	**2.25**	**0.00**	**1.93**	**0.00**	**-1.59**	**0.05**
*HALLMARK_UNFOLDED_PROTEIN_RESPONSE*	-1.45	0.11	**1.83**	**0.01**	**2.07**	**0.01**
*HALLMARK_UV_RESPONSE_DN*	**2.20**	**0.00**	-	-	**-2.21**	**0.00**
*HALLMARK_XENOBIOTIC_METABOLISM*	**1.81**	**0.01**	-	-	**-1.95**	**0.00**

### Protein-protein interactions network modeling and functional characterization

**Fig. 3:**
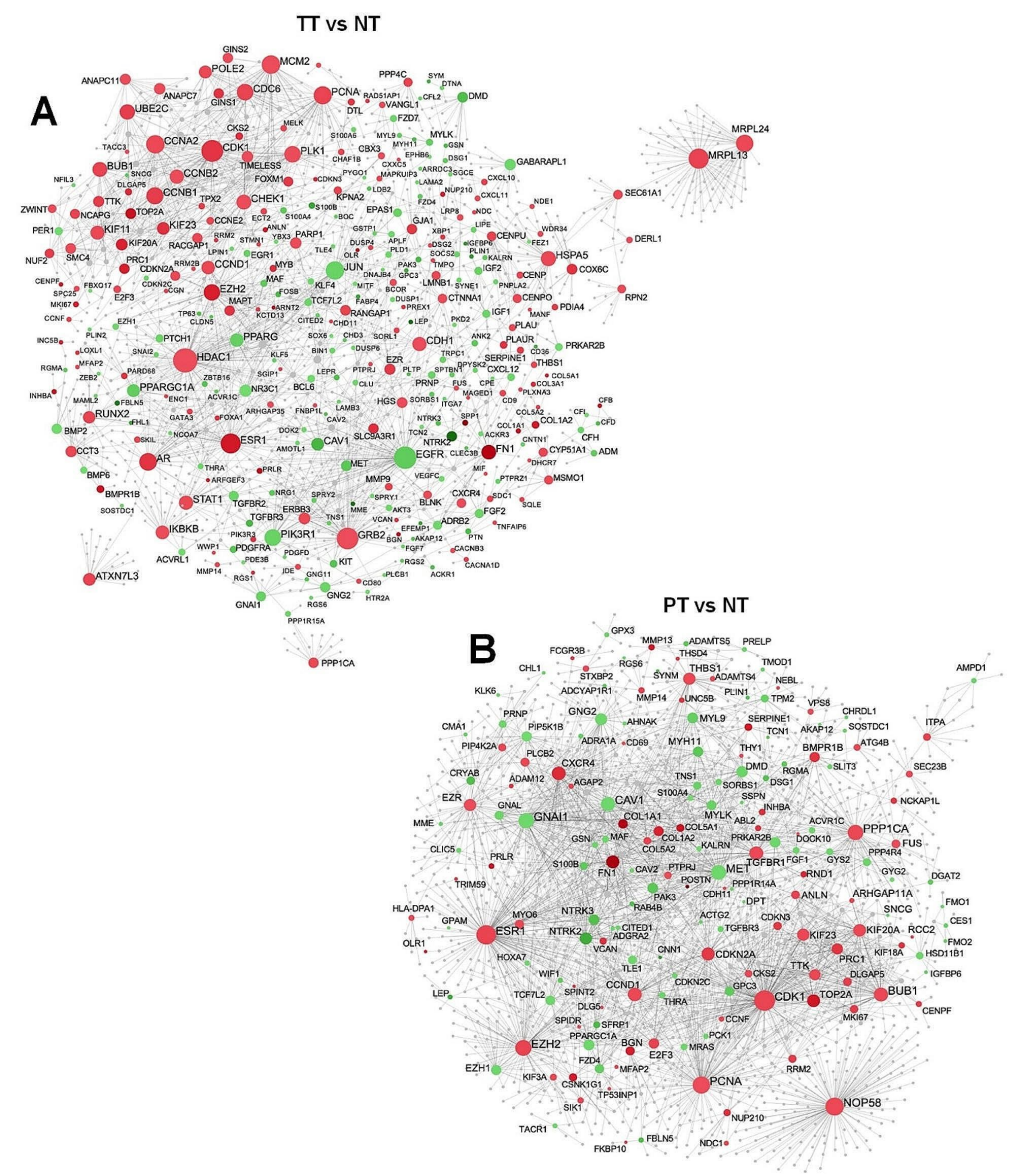
Protein-Protein Interactions between DEGs in early-stage IDC samples. PPI network analysis of DEGs identified in TT compared to NT (**A**) and PT compared to NT (**B**). Hub nodes were identified based on degree value, dependent on the number of connections to other nodes, and betweenness value, based on the number of shortest paths going through a node. Bigger nodes are hubs of the network. Green and red color of nodes are related to the expression of genes, down- and upregulated DEGs respectively. Grey nodes are genes that are not present in our data but are part of the PPI network

To identify key nodes of PT, interactions between proteins encoded by identified DEGs were performed by first-order Protein-Protein Interactions (PPI) networks based on STRING database. DEGs of the two comparisons TT vs. NT, and PT vs. NT were mapped, and subnetworks generated were analyzed and showed in Fig. 3 as subnetworks A and B, respectively. Subnetwork A contained 1447 nodes, 2456 edges, and 312 seeds (Fig. 3A); and subnetwork B contained 1834 nodes, 3051 edges, and 168 seeds (Fig. 3B). In addition, the subnetwork from the DEGs obtained from the comparison between PT and TT (subnetwork C) was generated and showed 907 nodes, 1208 edges, and 80 seeds (Figure S1).

To determine the functional significance of nodes connected in the subnetworks A and B, the three most relevant regulatory modules from the PPI networks were detected by WalkTrap algorithm and KEGG pathways enrichments of each module were carefully examined (Table 3). On the one hand, the nodes included in module 1 of the subnetwork A were associated with *Cell cycle*, *Cellular senescence*, *p53 signaling pathway*, *Viral carcinogenesis*, and *Pathways in cancer*; on the other hand, *ErbB signaling pathway*, *EGFR tyrosine kinase inhibitor resistance*, *Focal adhesion*, *Ras signaling pathway*, and *Proteoglycans in cancer* in module 2; and finally, *Transcriptional misregulation in cancer*, *Thyroid hormone signaling pathway*, *Pathways in cancer*, *Signaling pathways regulating pluripotency of stem cells*, and *Adherens junction* in module 3. In subnetwork B, *Cell cycle*, *Viral carcinogenesis*, *Cellular senescence*, *p53 signaling pathway*, and *Steroid hormone biosynthesis* KEGG pathways were found in module 1; *Focal adhesion*, *Ras signaling pathway*, *Proteoglycans in cancer*, *PI3K-Akt signaling pathway*, and *Regulation of actin cytoskeleton* in module 2; and *TGF-beta signaling pathway*, *Hippo signaling pathway*, *Cytokine-cytokine receptor interaction*, *Signaling pathways regulating pluripotency of stem cells*, and *Steroid hormone biosynthesis* in module 3.

Furthermore, the nodes included in subnetwork C (supplementary data), were involved in *ErbB signaling pathway*, *EGFR tyrosine kinase inhibitor resistance*, P*hospholipase D signaling pathway*, F*ocal adhesion*, and *Jak-STAT signaling pathway* in module 1; *Endocrine resistance*, *Breast cancer*, *Estrogen signaling pathway*, *Thyroid hormone signaling pathway*, and *Vasopressin-regulated water reabsorption* in module 2; and module 3 was associated to *Steroid hormone biosynthesis*, *Folate biosynthesis*, *Arachidonic acid metabolism*, *Metabolism of xenobiotics by cytochrome P450*, and *Chemical carcinogenesis* (Table S3).

**Table 3:** Highly connected modules identified at PPI networks and enrichment analysis of each module

Subnetwork A (TT vs. NT)			
Module	Genes	KEGG pathways	*p*-value
**Module 1**	ANLN, BUB1, CCNA2, CCNB1, CCNB2, CCND1, CCNE2 CDK1, CDKN2A, CDKN2C, CDKN3, CKS2, DLGAP5, E2F3, ECT2, FOXM1, KIF11, KIF20A, KIF23, MELK, MKI67, NCAPG, NUF2, PARP1, PLK1, PRC1, RACGAP1, RRM2, RRM2B, SMC4, SNCG, SPC25, TOP2A, TP63, TPX2, TTK, ZWINT	Cell cycle Cellular senescence p53 signaling pathway Viral carcinogenesis Pathways in cancer	7.75E-67 8.59E-26 3.81E-23 3.56E-19 1.25E-12
**Module 2**	ARHGAP35, BLNK, CAV1, CAV2, CDH1, CLEC3B, CTNNA1, EGFR, ERBB3, FNBPIL, GRB2, KALRN, KIT, MET, MME, MMP9, NRG1, PDGFD, PIK3R1, PIK3R3, PREX1, PTPRJ, SGIP1, SORBS1, SPRY1 SPRY2, TNS1, VEGFC	ErbB signaling pathway EGFR tyrosine kinase inhibitor resistance Focal adhesion Ras signaling pathway Proteoglycans in cancer	2.95E-47 1.13E-39 1.68E-36 3.57E-36 2.08E-28
**Module 3**	ACVR1C, AMOTL1, AR, CDH11, CDH3, ESR1, EZH1, EZH2, FOXA1, HDAC1, NCOA7, NR3C1, PPARG, PPARGC1A, RUNX2, SKIL, SNAI2, SOX6, STMN1, VTCF7L2, THRA, TLE4, ZBTB16, ZEB2	Transcriptional misregulation in cancer Thyroid hormone signaling pathway Pathways in cancer Signaling pathways regulating pluripotency of stem cells Adherens junction	1.02E-14 1.30E-11 4.54E-11 1.91E-08 2.78E-07
**Subnetwork B (PT vs. NT)**			
**Module**	**Genes**	**KEGG pathways**	**p-value**
**Module 1**	ANLN, ARHGAP11A, BUB1, CCND1, CCNF, CDK1, CDKN2A, CDKN2C CDKN3, CENPF, DLGAP5, E2F3, KIF18A, KIF20A, KIF23, MKI67, PRC1, RCC2, RRM2, SNCG, TOP2A, TTK	Cell cycle Viral carcinogenesis Cellular senescence p53 signaling pathway Steroid hormone biosynthesis	2.27E-77 9.98E-21 1.51E-16 1.05E-15 8.53E-07
**Module 2**	ADGRA2, CAV1, CAV2, CDH11, DLG5, DOCK10, FN1, GSN, KALRN, MET, NTRK2, NTRK3, PAK3, PRKAR2B, PTPRJ, RAB4B, S100A4, SORBS1, SPINT2, TCN1 TNS1, VCAN	Focal adhesion Ras signaling pathway Proteoglycans in cancer PI3K-Akt signaling pathway Regulation of actin cytoskeleton	8.44E-42 1.97E-34 1.18E-30 4.22E-26 4.45E-25
**Module 3**	BMPR1B, CHRDL1, INHBA, RGMA, SOSTDC1, TFGBR1, TGFBR3	TGF-beta signaling pathway Hippo signaling pathway Cytokine-cytokine receptor interaction Signaling pathways regulating pluripotency of stem cells Steroid hormone biosynthesis	2.91E-51 1.63E-16 4.34E-16 2.84E-10 8.09E-10

TT: Tumoral Tissue, PT: Peritumoral Tissue, and NT: Non-tumoral Tissue

### Identification of hub nodes in peritumoral tissue and their correlation with the prognosis of early-stage invasive ductal breast carcinoma

**Fig. 4:**
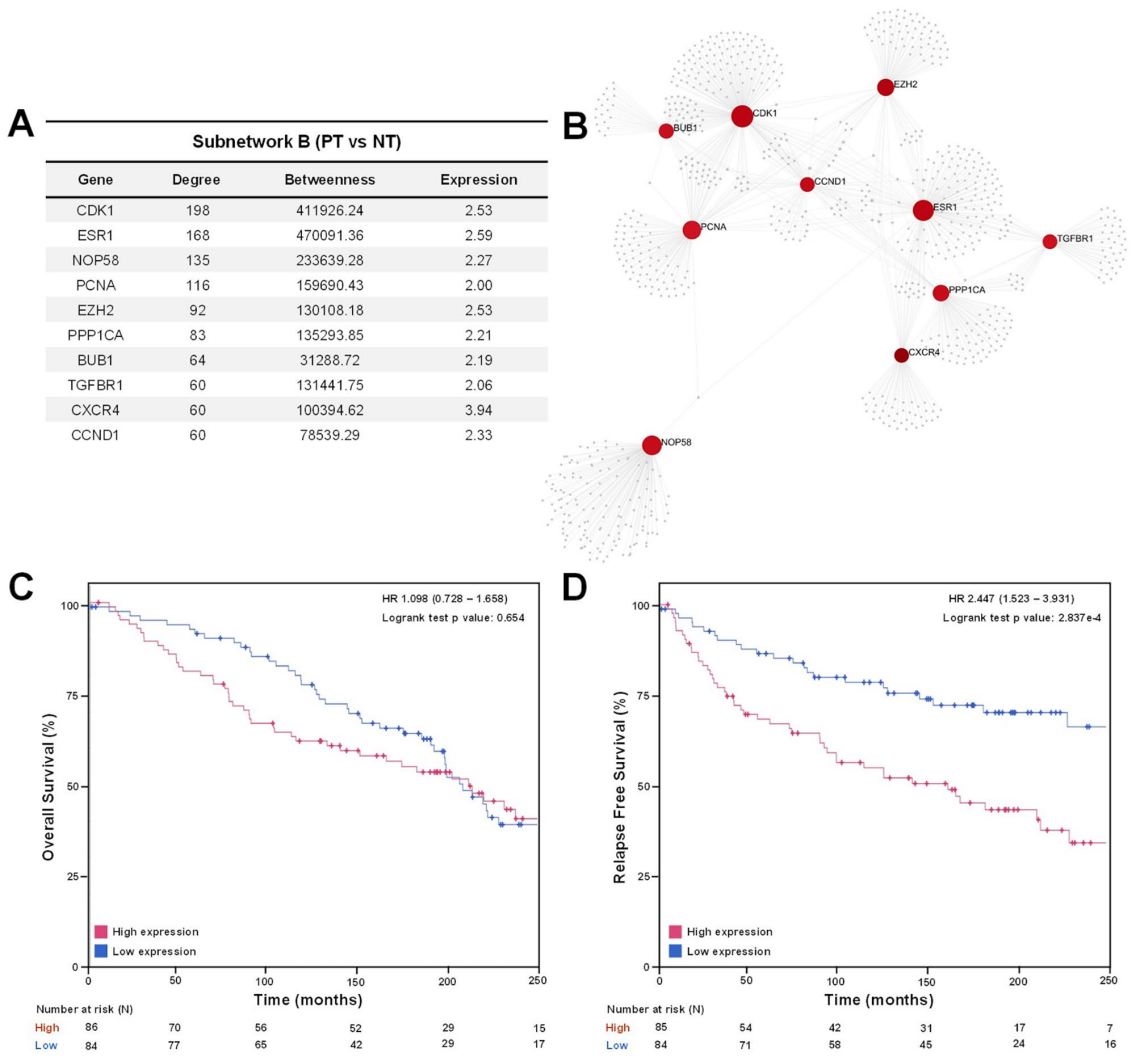
Main hub nodes identification of subnetwork B and association with OS and RFS. The top ten hub nodes of subnetwork B (**A**) interactions were mapped through PPI network analysis (**B**). Kaplan-Meier analysis of OS and RFS was performed to validate the relevance of the gene signature (including CDK1, PCNA, EZH2, and BUB1) in other IDC patients. OS (**C**) and RFS (**D**) analysis were analyzed through METABRIC database where blue indicates patients with low gene expression and red indicates patients with high gene expression

The most interactive upregulated nodes (CDK1, ESR1, NOP58, PCNA, EZH2, PPP1CA, BUB1, TGFBR1, CXCR4, and CCND1) of subnetwork B are shown in Fig. 4A, taking a count degree and betweenness, as well as their expression. Additionally, these hub nodes interaction was mapped obtaining a subnetwork that contained 822 nodes, 1026 edges, and 10 seeds (Fig. 4B).

To assess the relevance of the most hub nodes identified in subnetwork B, PT versus NT, including CDK1, ESR1, NOP58, PCNA, EZH2, PPP1CA, BUB1, TGFBR1, CXCR4, and CCND1, the analysis of overall survival (OS) and relapse-free survival (RFS) were analyzed by Kaplan-Meier method. As shown in Table S4, high mRNA expression of CDK1, PCNA, EZH2, BUB1, and CXCR4 in tumor was significantly associated with lower RFS. Only patients with high expression of ESR1 and BUB1 in tumor tissue showed a worse prognosis in terms of overall survival (OS) compared to those with low expression (Table S5). Furthermore, focusing on the most promising statistically significant candidates, CDK1, PCNA, EZH2, and BUB1, a gene signature enriched in PT was identified that did not show a significant association with poor OS (*p* = 0.654) but showed a strongly significant reduced RFS (*p* = 2.837e-4) in events in untreated luminal breast cancer patients (Fig. 4C and D, respectively).

**Fig. 5:**
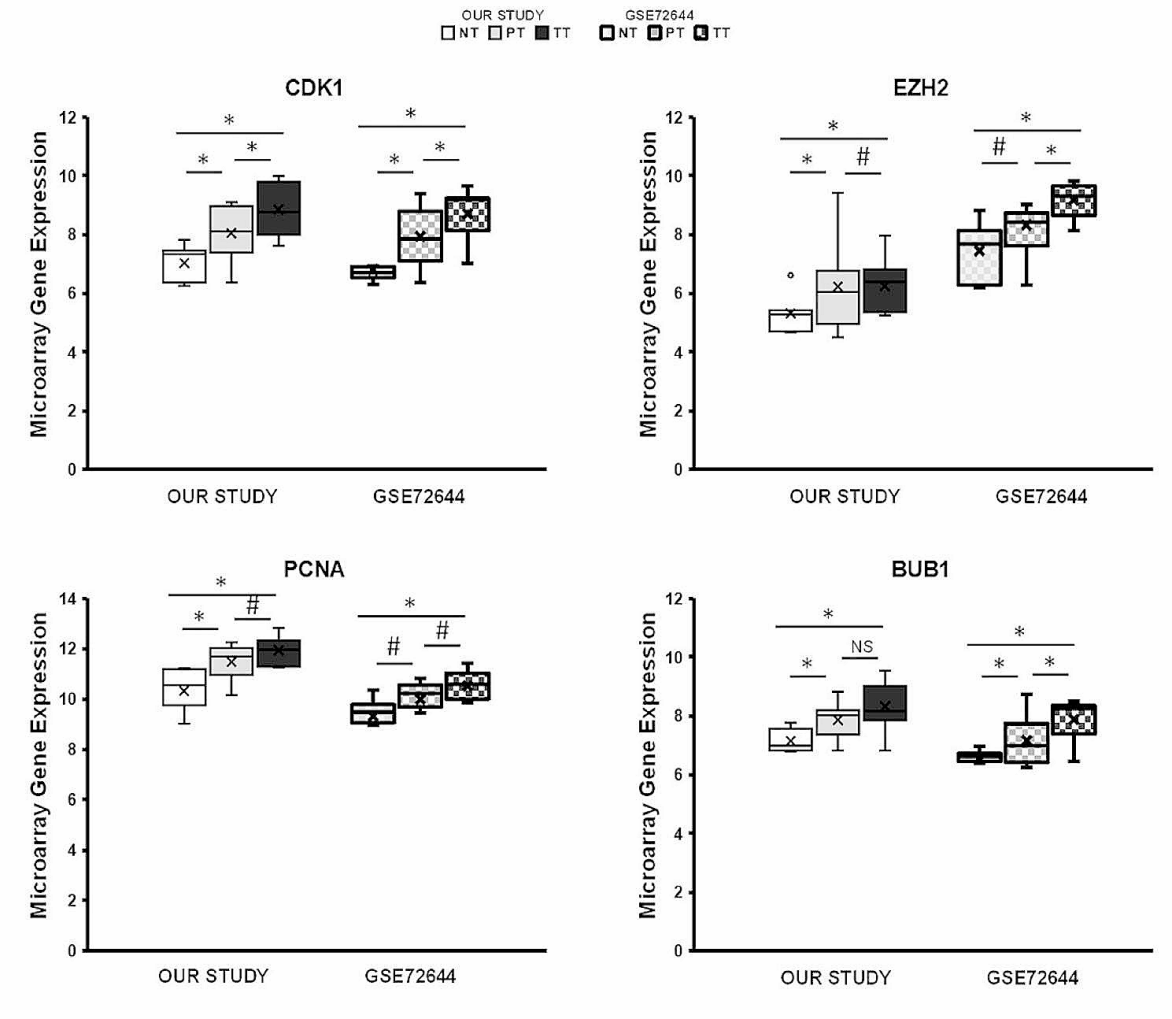
Comparison of the most relevant hub nodes mRNA expression with another cohort (GSE72644). Each box represents the interquartile range (25th to 75th percentiles), with the central horizontal line indicating the median value, and the whiskers representing the minimum and maximum values (*n* = 8). Student’s t-test were performed to determine the significance between the experimental groups. Statistical significance was set at * *p* < 0.05 and # *p* < 0.1

**Fig. 6:**
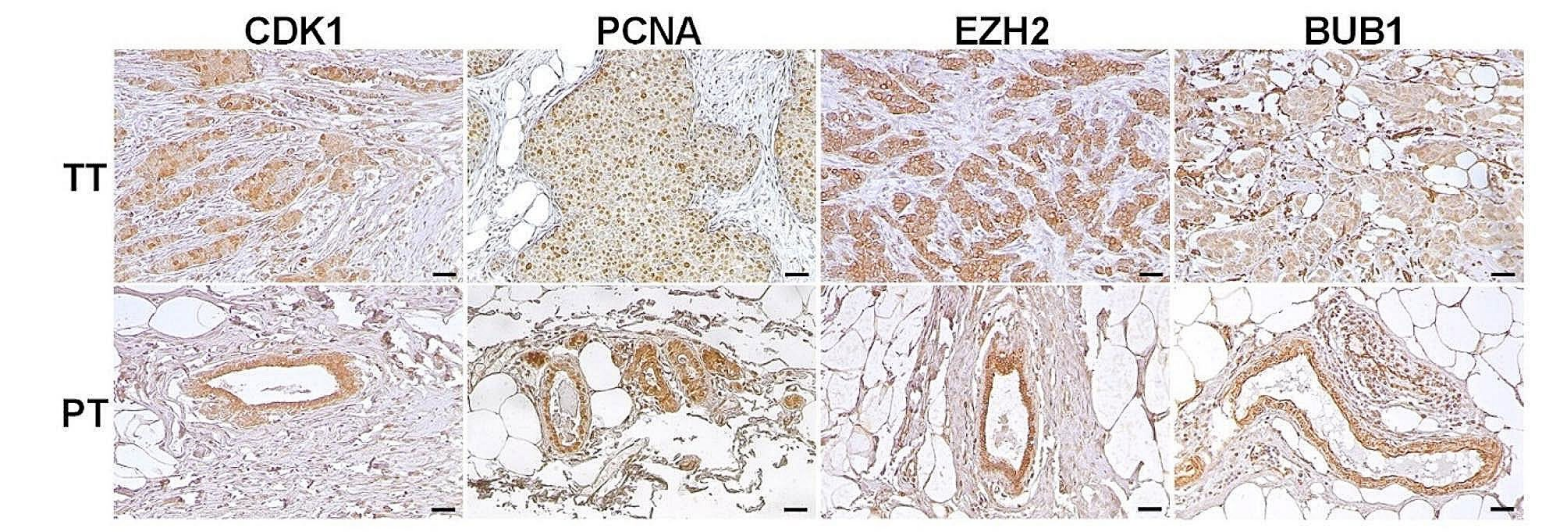
Immunohistochemistry analysis of gene-signature candidates in early-stage IDC patients. Representative images (20X) of CDK1, PCNA, EZH2 and BUB1 (from left to right) in tumoral (top) and peritumoral (bottom) tissues. Scale bars: 50 μm

Finally, the mRNA expression of the gene signature candidates, including CDK1, PCNA, EZH2 and BUB1, was analyzed individually using the database of another study (GSE72644). Consistent with our findings, the transcriptional expression levels of all genes were statistically significantly higher in PT compared to NT (Fig. 5). Microarray mRNA expression results were further validated in own cohort by RT-qPCR (Figure S2). In addition, the presence of the genes identified in the signature was analyzed by immunohistochemistry and found to be predominantly detected in ductal mammary epithelial cells (Fig. 6).

## Discussion

In this study, peritumoral tissue, spatially located between non-tumoral and tumoral tissues, has been explored in early-stage invasive ductal carcinomas, providing essential information to find prognostic biomarkers. Peritumoral tissue displays alterations in pathways related to proliferation, inflammation, and extracellular organization. In fact, we have identified upregulated key genes of cell cycle and cell progression in comparison with non-tumoral distant tissue, suggesting a proliferative phenotype transition in the peritumoral tissue that could be a driver of cancer relapse.

For many years, it has been established that tumor tissue cells, including breast cancer cells, possess distinct properties that set them apart from non-tumor tissue [24]. These properties include the sustained activation of proliferative signaling, evasion of growth suppressors, resistance to cell death, local invasion, and the ability to metastasize, all of which collectively drive cancer growth and progression [24]. As expected, in line with the findings of Vishnubalaji et al., our study also detected an enrichment of signaling pathways related to cell proliferation and interactions with the extracellular matrix in tumoral tissue as compared to non-tumoral tissue [25]. Notably, some of the highly upregulated genes observed play pivotal roles in these processes, such as HIST1H2BM, TSPAN1, TRPS1, MMP13, and FN1.

In this scenario, some recent studies propose that non-tumoral tissue located closer to the tumor, also called peritumoral tissue, displays unique molecular traits distinct from both the tumoral tissue itself and more distant non-tumoral tissue, evidencing the existence of an own entity for this peritumoral tissue [11, 13, 26]. Our transcriptomic results, comparing peritumoral and tumoral tissues, have shown an increased expression of genes implicated in crucial pathways such as the PPAR, the Adipocytokine, JAK-STAT, and AMPK signaling pathways in the area close to the tumor. In concordance with our findings, some studies reported that peritumoral tissue in breast cancer showed transcriptomic alterations caused by tumor proximity and adipose tissue influence [11, 13, 27, 28]. The pathways identified in the present study are known to play a significant role in adipogenesis, and the metabolic processes associated with adipose tissue, which is essential for the development and progression of breast cancer [28, 29]. Consistently, our data reveal a promising role of peritumoral tissue to recognize the early warning signs of relapse.

Leaving aside tumoral tissue, as demonstrated by Abdalla et al., our results also evidence how the molecular characteristics of distant and adjacent non-tumoral tissues are conditioned by proximity to the tumor [8]. In our study, we observed a heightened activity and an alteration of cell proliferation and tissue development of peritumoral tissue compared to non-tumoral tissue, with an enrichment of AGE-RAGE, p53, cell cycle, cellular senescence and Hedgehog signaling pathways, as well as ECM-receptor pathway and upregulation of genes involved in extracellular matrix remodeling, all of which are implicated in cancer hallmarks processes. These results agree with other findings that explore the influence of breast cancer cells on non-cancerous epithelial mammary gland cells MCF10A [30, 31], supporting the pre-malignant phenotype of peritumoral tissue. Furthermore, examining the functions of gene products using Gene Ontology (GO) terms, a strong association between the genes differentially expressed in peritumoral tissue and specific biological processes were found. These processes include cell motility, negative regulation of cell adhesion, and an enrichment of genes actively involved in extracellular organization. Within this context, it is noteworthy that Guo et al. observed the induction of an EMT-like phenotype in non-cancerous epithelial mammary gland cells, MCF10A, when were exposed to breast cancer cell-conditioned medium [32]. Similarly, Hwa Jo et al. reported an increased proliferative and colony-forming capacities of MCF10A cells when co-cultured with various breast cancer cell lines, aligning with our own findings that emphasize the influence of tumor proximity on biological processes associated with the positive regulation of the cell cycle and division [30].

As is widely known, tumor bulk establishes communication with the neighboring tissue, promoting an optimal environment to tumor development and resulting in significant alterations in both the tumor itself and its immediate surroundings [8, 11, 28, 33]. Analyzing protein-protein interaction (PPI) networks, we emphasized the study of gene modules and their interactions to understand the complexity of these transformations [19, 34]. In fact, we found how proximity to the tumor, comparing peritumoral and non-tumoral tissues, leads to the alteration of gene modules involved in signaling pathways related to cell proliferation and differentiation. Our findings reveal an alteration of a module, as well as in biological processes, implicated in the TGF-beta and Hippo signaling pathways, which Ye et al. previously identified as cooperative factors in driving carcinogenesis and metastasis in sarcomas [35]. This fact suggests that Hippo and TGF-beta axis could define potential targets for investigating the progression and recurrence, as well as for pharmaceutical intervention in breast cancer. Additionally, these results are supported by the enrichment of other pathways related to extracellular matrix remodeling and regulation of cell adhesion, two fundamental processes in the metastasis cascade [36, 37]. Therefore, our findings indicate an expression profile shift away towards one that supports a cancer profile.

Importantly, in contrast to breast non-tumoral tissue, peritumoral, as well as tumoral tissue, exhibits a prominent PPI module of altered genes associated with cell cycle, cellular senescence, and the p53 signaling pathway. According to our work, these findings underscore the altered cell proliferation of peritumoral tissue. Furthermore, when comparing peritumoral to non-tumoral tissues, some of the genes within this highly upregulated module, including BUB1, CCND1, and CDK1, also serve as central nodes in the Protein-Protein Interaction (PPI) network, that implies their critical roles in regulating the observed alterations in breast peritumoral tissue. These genes, along with other observed central nodes as PCNA, participate in various cellular processes in breast cancer, encompassing the regulation of the cell cycle, DNA replication, and cell division [38–43].

Highlighting the top ten upregulated nodes of the PPI subnetwork that compares peritumoral and non-tumoral tissues, our results showed a high interaction between most of them involving cell cycle, inflammation, and proliferative phenotype processes. In addition to the main nodes mentioned, BUB1, CCND1, CDK1, and PCNA, other studies also show the importance of NOP58, EZH2 and PPPC1A in tumorigenesis [44–46]. All these genes are interconnected in a complex network that ensures the correct control of cell division and genomic stability, emphasizing CDK1, as master regulator [39]. CDK1, CCND1, and PCNA play distinct roles in different phases of cell cycle and have been identified as pivotal genes in the development and progression of different cancer types, including colon, liver, and gynecological tumors [47–49]. In addition, several studies have demonstrated that CDK1, through phosphorylation of BUB1, EZH2 and PPP1CA, alters cell cycle regulation, chromosome segregation, epigenetic control, and DNA replication, leading to cell dysfunction and diseases such as cancer [41, 50, 51]. Complementarily, TGFBR1, another central hub node upregulated in peritumoral tissue compared to non-tumoral tissue, is the central propagator of TGF-beta signaling. The TGF-beta/TGFBR1 signaling pathway can act as a tumor suppressor or promote tumor progression, inducing G1-phase arrest by elevating the expression of cell cycle inhibitors in early-stage tumors or facilitating epithelial-to-mesenchymal transition (EMT) during the metastatic process, respectively [52]. However, consistent with the findings observed in our study, Gal et al. reported that prolonged exposure to TGFbeta suppresses both Smad and non-Smad signaling in mammary epithelial cells leading EMT and inhibiting growth arrest and apoptosis [53].

Finally, Chu et al. observed that early-stage ER + breast cancer tumors with elevated levels of CXCR4, another central hub node found in peritumoral tissue, are more likely to experience disease recurrence [54]. CXCR4 also contributes to immune suppression, supports tumor growth, and has the potential to chemoattract cancer cells to organs that produce its ligand, CXCL12, where cancer cells can establish secondary tumors [55, 56]. Therefore, here we define a gene signature in peritumoral tissue that could predict disease recurrence in early-stage breast cancer.

Taking all together, our results must be interpreted within the context of a significant estrogenic impact on breast cancer. ESR1 was identified as the second hub node in peritumoral versus non-tumoral tissues comparison, due to its high degree in the PPI network, observation that aligns with the characteristics of the study cohort. It is worthy to note that this cohort comprises patients with an early-stage IDC which have been pathologically classified as ER+. Many authors, including results from our laboratory, have demonstrated the carcinogenic effects of estrogens in hormone-dependent tissues, as in the case of the mammary gland [57–60]. These effects are guided, mainly, through the binding of estrogens to their receptor, ESR1, thus transactivating the gene expression of many genes related to cell proliferation and survival [61]. The fact that peritumoral tissue presents ESR1 upregulated in comparison with non-tumoral tissue means that the peritumoral tissue is more predisposed to suffer the effects of circulating estrogens, placing the results of this research in an estrogen-dependent scenario.

In this study, we further examined the clinical significance of a four-gene signature (comprising CDK1, PCNA, EZH2, and BUB1) that may be associated with relapse events in untreated luminal breast cancer patients. Our findings revealed a negative impact of these cell cycle-related genes on relapse-free survival (RFS) in patients whose tumors exhibited elevated expression levels of these four hub genes. Consequently, these results underscore the relevance of these genes in determining the clinical outcomes of the disease. Moreover, the study suggests that alterations in peritumoral tissue could serve as early indicators, detectable during routine checks, potentially preventing the onset of new tumors.

## Conclusions

Our study characterizes breast peritumoral tissue in depth, focused on differences with non-tumoral tissue, providing clues on the changes that tumor signaling could cause in patients with early-stage breast cancer. We have identified four genes, CDK1, PCNA, EZH2, and BUB1, that are overexpressed in peritumoral tissue compared to non-tumoral tissue. We therefore propose that the use of these genes, either individually or in combination as a signature, could help to predict local relapse. Therefore, the presence of these genes in the tissue remaining after surgical and/or radiotherapeutic treatment could drive the initial changes related to the malignant phenotypic transformation. Further research and clinical studies are needed to fully establish the role of peritumoral tissue, and the oncogenic significance of the identified hub nodes in regular check-ups for patients with invasive ductal breast cancer. Overall, our results highlight the value of this peritumoral tissue as a potential source of new biomarkers for early detection of relapse and improvement in invasive ductal carcinoma patient’s prognosis.

### Electronic supplementary material

Below is the link to the electronic supplementary material.

Supplementary Material 1

Supplementary Material 2

## Data Availability

The datasets generated and/or analysed during the current study are available in the supporting files.
