# Supplementary material for: Unraveling malignant phenotype of peritumoral tissue: transcriptomic insights into early-stage breast cancer
Source: Breast Cancer Res. 2024 Jun 3;26:89. doi: 10.1186/s13058-024-01837-2 (PMC11145834; doi:10.1186/s13058-024-01837-2)
Supplement: Supplementary file 2 — Supplementary Material 2 [file 13058_2024_1837_MOESM2_ESM.docx]

**Supplementary data**

**Table S1.**- Clinicopathological features of patients included in the study.

| **Age (years)** | **Stage** | **TNM** | **Molecular Subtype** |
| --- | --- | --- | --- |
| 72 | IA | pT1c pN0(sn) | Luminal A |
| 65 | IIA | pT2 pN0(sn) | Luminal A |
| 54 | IB | pT1c pN1mi(sn) | Luminal A |
| 29 | IIB | pT2 pN1a(sn) | Luminal A |
| 65 | IA | pT1b pN0(i+)(sn) | Luminal A |
| 53 | IA | pT1c pN0(sn) | Luminal A |
| 76 | IA | pT1c pN0(sn) | Luminal A |
| 67 | IA | pT1c pN0(sn) | Luminal B |
| 55 | IIA | pT1c pN1mi(sn) | Luminal A |
| 69 | IA | pT1c pN0(sn) | Luminal A |

**Table S2.**- Primers and conditions used for RT-qPCR.

| **Gene**  **Accession Number** | **Forward Primer (5′-3′)**  **Reverse Primer (5′-3′)** | **Annealing Temperature (°C)** |
| --- | --- | --- |
| **CDK1**  NM_001786.5 | AAACTACAGGTCAAGTGGTAGCC  TCCTGCATAAGCACATCCTGA | 60 |
| **PCNA**  NM_002592.2 | TGAAGCACCAAACCAGGAG  GAAGGCATCTTTACTACACAGC | 57 |
| **EZH2**  NM_004456.5 | GCCAGACTGGGAAGAAATCTG  TGTGCTGGAAAATCCAAGTCA | 60 |
| **BUB1**  NM_004336.5 | TGGGAAAGATACATACAGTGGGT  AGGGGATGACAGGGTTCCAAT | 60 |
| **18S**  NR_146119.1 | GGACACGGACAGGATTGACA  ACCCACGGAATCGAGAAAGA | 60 |


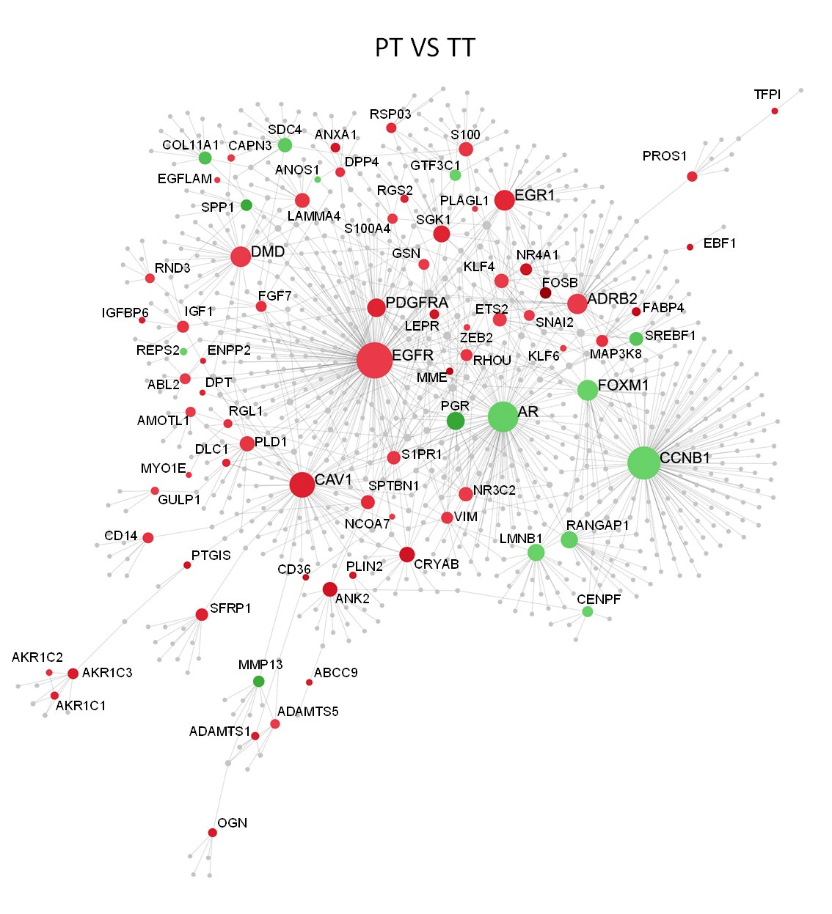


**Figure S1. Protein-Protein Interactions between DEGs in early-stage IDC-NOS samples.** PPI network analysis of DEGs identified in PT compared to TT. Hub nodes were identified based on degree value, dependent on the number of connections to other nodes, and betweenness value, based on the number of shortest paths going through a node. Bigger nodes are hubs of the network. Green and red color of nodes is related to the expression of genes, down- and upregulated DEGs respectively. Grey nodes are genes that are not present in our data but are part of the PPI network.

**Table S3.** Highly connected modules identified at PPI networks and enrichment analysis of each module.

| **Subnetwork C (PT vs TT)** | | | |
| --- | --- | --- | --- |
| **Module** | **Genes** | **KEGG pathways** | **p-value** |
| **Module 1** | AR, EGFR, KLF6, LEPR, MME, PDGFRA, RHOU, S1PR1 | ErbB signaling pathway  EGFR tyrosine kinase inhibitor resistance  Phospholipase D signaling pathway  Focal adhesion  Jak-STAT signaling pathway | 1.01E-44  2.14E-39  8.03E-26  1.14E-23  2.73E-23 |
| **Module 2** | ETS2, NCOA7, NR3C2, PGR, PLAGL1 | Endocrine resistance  Breast cancer  Estrogen signaling pathway  Thyroid hormone signaling pathway  Vasopressin-regulated water reabsorption | 3.41E-12  2.03E-10  2.79E-09  1.56E-08  1.93E-08 |
| **Module 3** | AKR1C1, AKR1C2, AKR1C3 | Steroid hormone biosynthesis  Folate biosynthesis  Arachidonic acid metabolism  Metabolism of xenobiotics by cytochrome P450  Chemical carcinogenesis | 1.30E-09  1.87E-06  0.00177  0.00257  0.00298 |

**Table S4.** Relapse-Free Survival results of the main hub nodes identified in subnetwork B.

| **Gene** | **High expression group** | |  | **Low expression group** | |  | **p-value** |
| --- | --- | --- | --- | --- | --- | --- | --- |
|  | **HR** | **95% CI** |  | **HR** | **95% CI** |  |  |
| **CDK1** | 1.612 | 1.173-2.214 |  | 0.620 | 0.452-0.852 |  | 3.28E-03 |
| **ESR1** | 1.187 | 0.864-1.630 |  | 0.842 | 0.613-1.157 |  | 0.280 |
| **NOP58** | 0.886 | 0.646-1.217 |  | 1.128 | 0.822-1.549 |  | 0.450 |
| **PCNA** | 1.474 | 1.073-2.024 |  | 0.679 | 0.494-0.932 |  | 0.0163 |
| **EZH2** | 1.795 | 1.306-2.466 |  | 0.557 | 0.405-0.766 |  | 6.60E-04 |
| **PPP1CA** | 1.229 | 0.895-1.687 |  | 0.814 | 0.593-1.118 |  | 0.200 |
| **BUB1** | 1.897 | 1.381-2.607 |  | 0.527 | 0.384-0.724 |  | 8.94E-05 |
| **TGFBR1** | 0.768 | 0.559-1.055 |  | 1.302 | 0.948-1.789 |  | 0.120 |
| **CXCR4** | 1.231 | 0.946-1.602 |  | 0.812 | 0.624-1.057 |  | 0.046 |
| **CCND1** | 0.980 | 0.714-1.346 |  | 1.020 | 0.743-1.400 |  | 0.902 |

HR= Hazard Ratio; CI=Confidence Interval

**Table S5.** Overall Survival results of the main hub nodes identified in subnetwork B.

| **Gene** | **High expression group** | |  | **Low expression group** | |  | **p-value** |
| --- | --- | --- | --- | --- | --- | --- | --- |
|  | **HR** | **95% CI** |  | **HR** | **95% CI** |  |  |
| **CDK1** | 1.076 | 0.828-1.396 |  | 0.930 | 0.716-1.207 |  | 0.582 |
| **ESR1** | 1.723 | 1.326-2.238 |  | 0.581 | 0.447-0.754 |  | 7.81E-05 |
| **NOP58** | 0.769 | 0.592-0.999 |  | 1.301 | 1.001-1.690 |  | 0.092 |
| **PCNA** | 0.995 | 0.766-1.291 |  | 1.005 | 0.775-1.305 |  | 0.968 |
| **EZH2** | 1.120 | 0.863-1.454 |  | 0.893 | 0.688-1.159 |  | 0.392 |
| **PPP1CA** | 0.877 | 0.676-1.139 |  | 1.140 | 0.878-1.479 |  | 0.326 |
| **BUB1** | 1.337 | 1.030-1.736 |  | 0.748 | 0.576-0.971 |  | 0.0281 |
| **TGFBR1** | 0.852 | 0.656-1.106 |  | 1.174 | 0.904-1.525 |  | 0.226 |
| **CXCR4** | 1.231 | 0.946-1.602 |  | 0.812 | 0.624-1.057 |  | 0.114 |
| **CCND1** | 1.101 | 0.848-1.430 |  | 0.908 | 0.699-1.178 |  | 0.466 |

HR= Hazard Ratio; CI=Confidence Interval

**Figure S2. Validation of the four-gene signature mRNA expression by RT-qPCR.** Each box represents the interquartile range (25^th^ to 75^th^ percentiles), with the central horizontal line indicating the median value, and the whiskers representing the minimum and maximum values. Student’s t-test were performed to determine the significance between the experimental groups. Statistical significance was set at * p < 0.05.
